# Design, synthesis and biological activity of selective hCAs inhibitors based on 2-(benzylsulfinyl)benzoic acid scaffold

**DOI:** 10.1080/14756366.2019.1651315

**Published:** 2019-08-11

**Authors:** Giulia Rotondi, Paolo Guglielmi, Simone Carradori, Daniela Secci, Celeste De Monte, Barbara De Filippis, Cristina Maccallini, Rosa Amoroso, Roberto Cirilli, Atilla Akdemir, Andrea Angeli, Claudiu T. Supuran

**Affiliations:** a Dipartimento di Chimica e Tecnologie del Farmaco, Sapienza University of Rome, Rome, Italy;; b Department of Pharmacy, “G. D’Annunzio”, University of Chieti-Pescara, Chieti, Italy;; c Centro Nazionale per il Controllo e la Valutazione dei Farmaci, Istituto Superiore di Sanità, Rome, Italy;; d Computer-aided Drug Discovery Laboratory, Faculty of Pharmacy, Department of Pharmacology, Bezmialem Vakif University, Fatih, Istanbul, Turkey;; e Neurofarba Department, Section of Pharmaceutical and Nutraceutical Sciences, Università degli Studi di Firenze, Sesto Fiorentino (Florence), Italy

**Keywords:** Carbonic anhydrase inhibitor, sulfoxide enantioseparation, carboxylic acid, molecular modelling

## Abstract

A large library of derivatives based on the scaffold of 2-(benzylsulfinyl)benzoic acid were synthesised and tested as atypical inhibitors against four different isoforms of human carbonic anhydrase (hCA I, II, IX and XII, EC 4.2.1.1). The exploration of the chemical space around the main functional groups led to the discovery of selective hCA IX inhibitors in the micromolar/nanomolar range, thus establishing robust structure-activity relationships within this versatile scaffold. HPLC separation of some selected chiral compounds and biological evaluation of the corresponding enantiomers was performed along with molecular modelling studies on the most active derivatives.

## 1. Introduction

Carbonic anhydrases (CAs, EC 4.2.1.1) are important metalloenzymes involved in the hydration of carbon dioxide, a quite simple reaction whose balance is important in many cellular and physiological processes, spanning from pH homoeostasis and respiration to biosynthetic pathways including lipogenesis, glucogenesis, and ureagenesis^1–3^.

Human CAs (hCAs) exist in fifteen isoforms, which possess different characteristics as catalytic activity, tissues distribution, cellular localisation (cytosol, mitochondria and cell membrane) and sensibility to inhibitors. The researches of the last years showed all the consequences of their altered activity, either in the case of excessive or deficient ones^4^^,^^5^. In this regard, investigations on synthetic and natural compounds have been done with the aim to discover new selective and potent inhibitors and/or activators, which could restore the normal functions of these enzymes^6–13^.

One of the most studied alterations has been observed in tumours, where some hCA isoforms appear overexpressed; the so-called tumour-related isoforms, hCA IX and XII which are transmembrane enzymes, participate along with cytosolic hCA II in the complex pH machinery which controls the *milieu* of hypoxic tumours, promoting drug resistance as well as proliferation, migration, invasion and metastasis of tumour cells^14–17^. To reverse this condition, efforts have been made to develop inhibitors that act effectively and selectively towards these targets.

**Figure 1.**
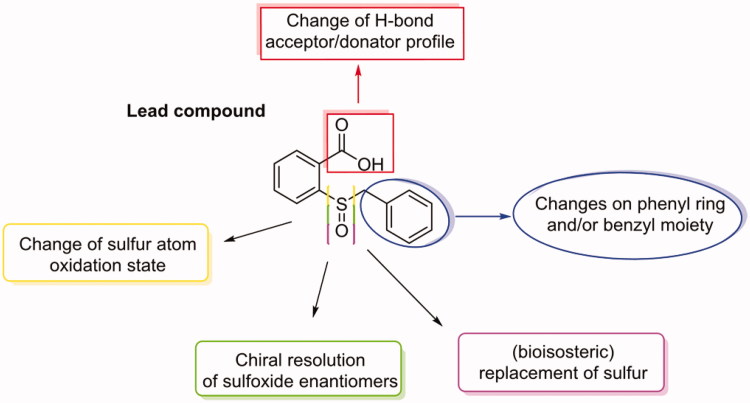
Changes performed on the 2-(benzylsulfinyl)benzoic acid scaffold.

The design of more molecules and scaffolds, and the obtaining of co-crystals of some of them with hCA enzymes (mostly with hCA II), prompted the discovery of four different mechanisms of inhibition for hCAs^18^^,^^19^. The most recently reported one was observed for the first time with 2(benzylsulfinyl)benzoic acid (Figure 1) co-crystallized with hCA II^20^. This compound showed an atypical mechanism involving the occupancy of a pocket next to the entrance of the active site, where it established some interactions holding the His64 residue in the “*out*” conformation. This amino acid, which exists in two conformations called “*in*” and “*out*”, acts as a proton-shuttle system transferring a proton from the zinc-coordinated water molecule to the environment, to reconstitute the catalytic active form. Interfering with this process (the rate-determining step of the entire catalytic cycle) is equal to block or strongly reduce the enzymatic activity, obtaining the enzyme inhibition. Our lead compound was inactive against hCA I and XII (*K*_i_ hCA I/XII >10 µM), while showed inhibitory activity in the low micromolar range towards both hCA II and IX, with a slight preference for the former (*K*_i_ hCA II = 0.15 µM, *K*_i_ hCA IX = 1.29 µM). With this in mind, we developed a novel series of derivatives based on the lead compound scaffold, pursuing some changes on its structure in order to improve activity and selectivity (Figure 1).

The carboxylic acid moiety (red in Figure 1) was replaced with methyl ester, amide, *N*-methyl amide, hydroxamic acid and ketone. The selected functionalities possess different hydrogen-bond acceptor/donator profiles if compared with the carboxylic acid one; furthermore, except for amide and hydroxamic acid moieties that still conserve a “deprotonatable” group, the others lack this feature whose importance has been in this way deepened.

The benzyl moiety (blue in Figure 1) was challenged through the insertion of substituents or by its entire substitution with benzoylmethyl and phenylacetic moieties as well as unsaturated C_3_–C_5_ alkyl chains. The aim of these changes was to understand the importance of the hydrophobic interactions with Phe231 and Asn232 that the phenyl ring established in the hCA II-inhibitor adduct. The addition of substituents on this phenyl ring changed the π-system charge distribution along with its hydro/lipophilicity. On the contrary, moving the phenyl group away from the sulphur atom with the addition of a carbonyl group (in the benzoylmethyl or phenylacetic derivatives) shed light on the importance of this system in that position and its conformational freedom. Attempts with unsaturated alkyl chains with increasing lengths have been done in order to evaluate if the presence of groups, able to give hydrophobic interactions, could work similarly to the phenyl ring. The role of sulphur atom was also challenged (yellow, green and violet in Figure 1), evaluating how both its oxidation state and its bioisosteric replacement affected the inhibitory activity. In the first case, we performed sulphide oxidation in order to obtain sulphinyl (sulfoxides) and sulphonyl (sulfones) derivatives. This synthesis was performed without chiral auxiliaries, obtaining sulfoxides in a mixture of enantiomers (racemate) that were resolved using a chiral HPLC system^21^^,^^22^ and individually tested; this permitted the evaluation of how chirality could affect inhibitory activity of these derivatives. On the contrary, the bioisosteric replacement of sulphur atom with oxygen and nitrogen ones or with methylene group has been done, unravelling the importance of that atom for the inhibitory activity (violet in Figure 1).

## 2. Chemistry and HPLC enantioseparation

**Scheme 1.**
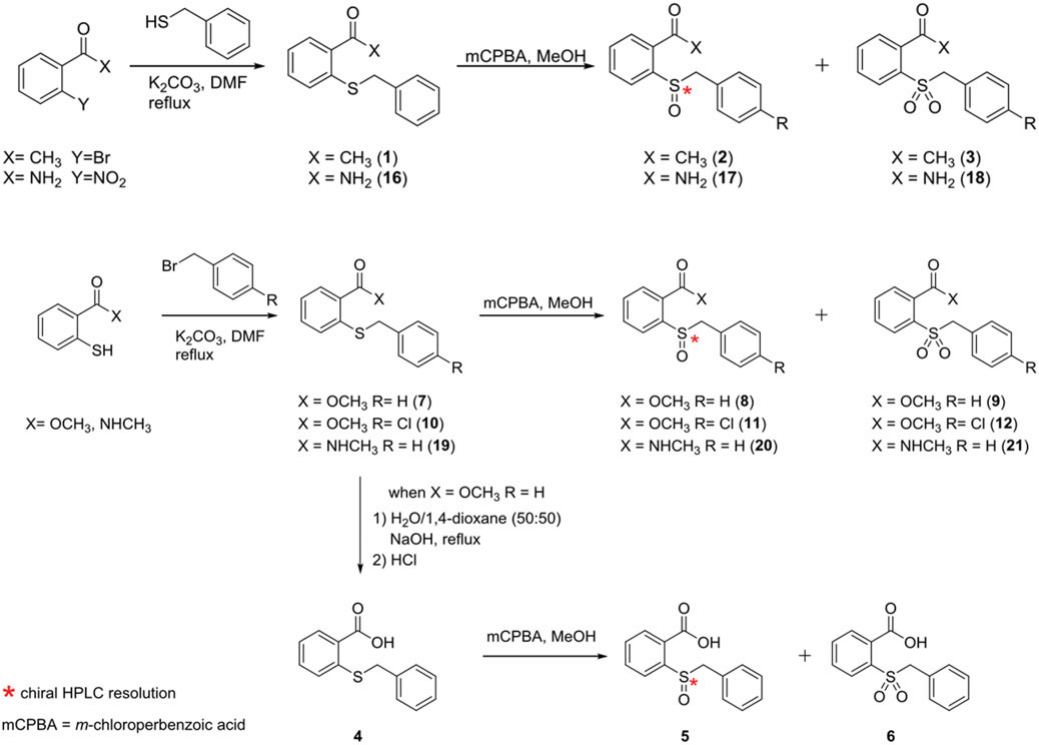
Synthesis and structures of compounds **1–12** and **16**–**21**.

**Scheme 2.**
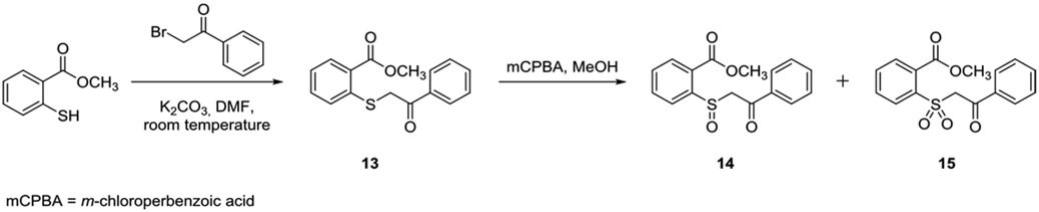
Synthesis and structures of compounds **13–15**.

**Scheme 3.**
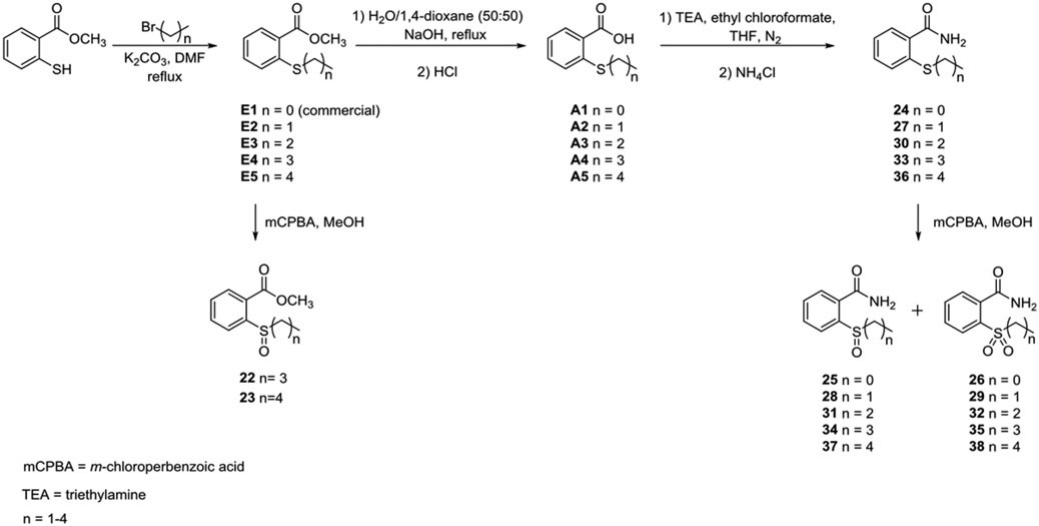
Synthesis and structures of compounds **22–38**.

**Scheme 4.**
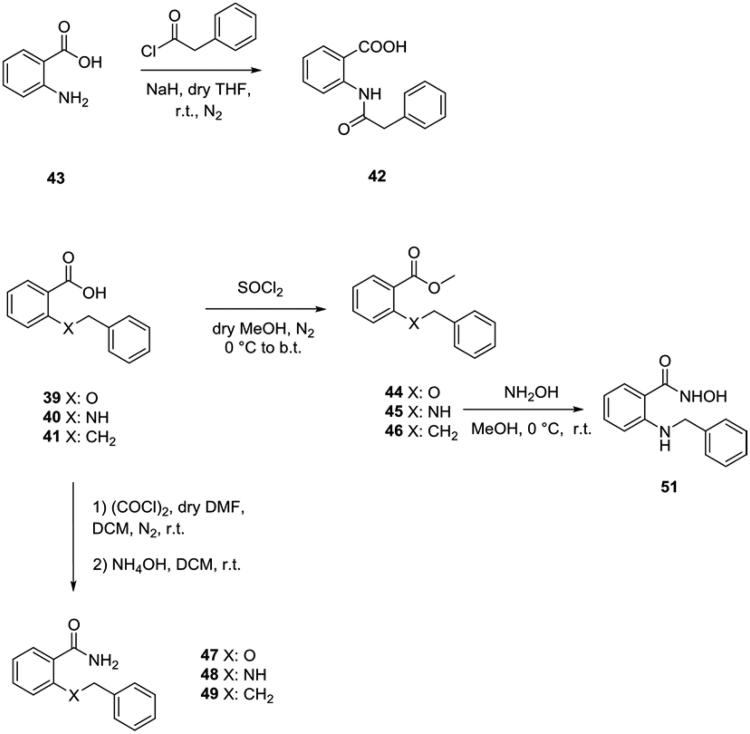
Synthesis of the amide **42**, esters **44–46**, hydroxyamide **51** and amides **47–49**.

In order to obtain the novel compounds, we followed the synthetic strategies reported in Schemes 1–4. For the synthesis of derivatives **1–3** and **16–18** we started from 1–(2-bromophenyl)ethan-1-one and 2-nitrobenzamide, respectively (Scheme 1); both of them (individually) underwent an aromatic nucleophilic substitution with benzyl mercaptan. The reactions were performed in *N,N’*dimethylformamide (DMF) at reflux, in the presence of K_2_CO_3_. The so obtained compounds (**1** and **16**) were treated in the next oxidative reaction with *meta*-chloroperbenzoic acid (mCPBA). Although many routes able to afford sulphur oxidation exist^23^, we have deliberately chosen this approach to obtain the two oxidation products, the sulfoxide and the sulphone, in the same reaction.

In fact, controlling the amount of oxidant added during the reaction we obtained both the species that were easily separated owing to the different chromatographic profiles. Compounds **7–12** and **19–21** were obtained with a nucleophilic substitution reaction between methyl 2-mercaptobenzoate and (un)substituted benzyl bromides, followed by the oxidative step. The carboxylic acid derivatives were obtained through the hydrolysis of compound **7** with aqueous 2 N sodium hydroxide (NaOH) in a mixture of equal amounts of water and 1,4-dioxane (50/50, *v*/*v*) to give **4**, that was subsequently oxidised to **5** and **6** (Scheme 1). Compounds **13**–**15** were synthesised performing the first step of nucleophilic substitution between methyl 2-mercaptobenzoate and benzoyl bromide at room temperature, with successive oxidation in the same conditions previously seen (Scheme 2). The synthetic pathway of derivatives **22**–**38** is reported in Scheme 3. Methyl 2mercaptobenzoate was reacted in the presence of bromoalkanes of increasing length, in DMF at reflux in the presence of K_2_CO_3_ to obtain intermediates **E2–E5** (**E1**, methyl 2-(methylthio)benzoate, was commercially available). Only two of the **E** intermediates bearing butyl and pentyl chain respectively (**E4** and **E5**), underwent the oxidative step obtaining the sulphinyl derivatives **22–23**. All the **E1**–**E5** intermediates were hydrolysed with aqueous 2 N sodium hydroxide in a mixture of equal amounts of water and 1,4-dioxane (50/50, *v*/*v*). After synthesis completion, the reactions were quenched with hydrochloric acid (HCl), giving the intermediates **A1–A5** (Scheme 3). The activation of the carboxylic acid group for the amide synthesis has been obtained using the mixed anhydride approach. In this regard, we used ethyl chloroformate in tetrahydrofuran (THF) under nitrogen atmosphere (N_2_) in the presence of triethylamine (Et_3_N) excess. Monitoring the reaction with thin layer chromatography (TLC) we detected the disappearance of the acidic intermediate, until the completion of anhydride formation. Then, ammonium chloride (NH_4_Cl) was added. The presence of the triethylamine excess involved the “de-blocking” of NH_3_ from NH_4_Cl, giving the final products (**24**, **27**, **30**, **33**, **36**).

Although chiral properties are often linked with the presence of a quaternary carbon atom binding different substituents, even the sulphur atom can have chiral behaviour under appropriate conditions.

The sulfoxides have a pyramidal structure where one vertex contains the sulphur electron pair, which can be considered as the fourth substituent bound to the sulphur atom. Therefore, if the two groups bound on the sulphinyl moiety are diverse, there is a chiral centre and the presence of two enantiomers. Differing from tertiary amines, which had low pyramidal inversion energy barrier, the sulfoxides possess energy barrier that allows the existence of two stable enantiomers, which can be resolved. Some sulfoxides (**5**, **8**, **11**, **17**, **20**) obtained in of the synthetic pathway shown in Schemes 1–4, were selected and mg-quantities of their pure enantiomeric forms easily isolated by semipreparative enantioselective HPLC on the 250 mm × 10 mm i.d. Chiralpak IC column using pure ethanol as a mobile phase^24^.

In compounds **39**–**53**, the sulphur atom characterising molecules **1**–**38** was replaced by an isosteric oxygen (**39**, **44**, **47**, **52**), nitrogen atom (**40, 42**, **43**, **45**, **48**, **50**, **51**, **53**), or alkyl chain (**46**, **41**, **49**). Compounds **39–41** and **43** (anthranilic acid) were commercially available and used as purchased.

Compound **42** was obtained starting from anthranilic acid, which was treated with sodium hydride (NaH) in dry THF. After 10 min, the phenylacetyl bromide was added at room temperature under nitrogen, giving the desired amide (Scheme 4). Compounds **44–46** were synthesised starting from the corresponding carboxylic acids **39**–**41**, which were treated with sulphonyl chloride (SOCl_2_) in boiling dry methanol to give the corresponding methyl esters (Scheme 4)^25^.

**Scheme 5.**
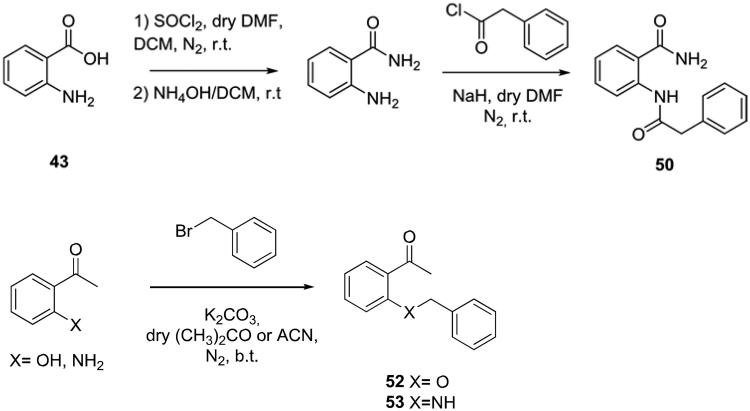
Synthesis of amide **50** and ketones **52–53**.

The reaction of the ester **45** with hydroxylamine hydrochloride in methanol at room temperature for 48 h gave the *N*-hydroxy benzamide **51**^26^. Compounds **47–49** were obtained converting the carboxylic acids **39–41** to the corresponding acyl chlorides by means of oxalyl chloride in the presence of a catalytic amount of dry DMF. Acyl chlorides were subsequently treated with ammonium hydroxide (NH_4_OH), to afford the desired amides (Scheme 4)^27^. The amide **50** was obtained by a different route (Scheme 5). At first, it was synthesised the amide from the anthranilic acid activated by SOCl_2_ in DMF at room temperature as previously reported. The addiction of NaH and phenylacetyl chloride in dry THF at room temperature let to the final amide **50**.

Finally, ketones **52** and **53** were easily obtained through the reaction of 2-hydroxy or 2-aminoacetophenone, respectively, with benzylbromide in the presence of K_2_CO_3_ in dry acetone or acetonitrile, respectively (Scheme 5).

## 3. Experimental protocols

### 3.1. General

Solvents were used as supplied without further purification. Starting materials and other chemicals were purchased by Sigma-Aldrich (Milan, Italy) and used in the syntheses and in the biological assays without further purification. All synthesised compounds have been fully characterised by analytical and spectral data. Column chromatography was carried out using Sigma-Aldrich^®^ silica gel (high purity grade, pore size 60 Å, 200–425 mesh particle size). Analytical thin-layer chromatography was carried out on Sigma-Aldrich^®^ silica gel on TLC aluminium foils with fluorescent indicator 254 nm. Visualisation was carried out under UV irradiation (254 and 365 nm). ^1^H NMR spectra were recorded on a Bruker AV400 (^1^H: 400 MHz, ^13 ^C: 101 MHz). Chemical shifts are quoted in ppm, based on appearance rather than interpretation, and are referenced to the residual non deuterated solvent peak. Missing signals must be attributed to overlapping peaks. Infrared spectra of the most representative compounds were recorded on a Bruker Tensor 27 FTIR spectrometer equipped with an attenuated total reflectance attachment with internal calibration.

Absorption maxima (ν_max_) are reported in wavenumbers (4000–400 cm^−1^). Elemental analyses for C, H, and N were recorded on a Perkin-Elmer 240 B microanalyzer obtaining analytical results within ± 0.4% of the theoretical values for all compounds. All melting points were measured on a Stuart^®^ melting point apparatus SMP1 and are uncorrected (temperatures are reported in °C). Where given, systematic compound names are those generated by ChemBioDraw Ultra^®^ 12.0 following IUPAC conventions. Mass spectra on the most representative compounds were performed on a LCQ (Thermo Finnigan) ion trap mass spectrometer (San Jose, CA, USA) equipped with an electrospray ionisation (ESI) source. The capillary temperature was set at 300 °C and the spray voltage at 4.25 kV. The fluid was nebulised using nitrogen (N_2_) as both the sheath gas and the auxiliary gas. All the characterisation data for each compound were reported as Supplemental material.

### 3.2. Enzyme inhibition assays

An Applied Photophysics stopped-flow instrument has been used for assaying the CA catalysed CO_2_ hydration activity^28^. Phenol red (0.2 mM) has been used as indicator, working at the absorbance maximum of 557 nm, with 20 mM Hepes (pH 7.5, for α-CAs) as buffer and 20 mM NaClO_4_ (for maintaining constant the ionic strength), following the initial rates of the CA-catalysed CO_2_ hydration reaction for a period of 10–100 s. The CO_2_ concentrations ranged from 1.7 to 17 mM for the determination of the kinetic parameters and inhibition constants. In particular, CO_2_ was bubbled in distilled deionised water for 30 min till saturation. A CO_2_ kit (Sigma, Milan, Italy) was used to measure the concentration in serially diluted solutions from the saturated one at the same temperature. For each inhibitor at least six traces of the initial 5–10% of the reaction have been used for determining the initial velocity. The uncatalyzed rates were determined in the same manner and subtracted from the total observed rates. Stock solutions of inhibitor (1 µM) were prepared in distilled-deionised water and dilutions up to 0.1 nM were done thereafter with the assay buffer. Inhibitor and enzyme solutions were preincubated together for 15 min at room temperature prior to assay to allow for the formation of the E-I complex or for the eventual active site mediated hydrolysis of the inhibitor. The inhibition constants were obtained by non-linear least-squares methods using PRISM 3 and the Cheng-Prusoff equation^29^, and represent the average from at least three different determinations. All recombinant CA isoforms were obtained in-house as previously reported^30^^,^^31^.

### 3.3. Molecular modelling studies

The three-dimensional structures of all ligands were prepared in their lowest energy conformation using the MOE software package (v2019.01, Chemical Computing Group, Inc, Montreal, Canada). The sulphonamide nitrogen atoms of the ligands were assigned a negative charge (R-SO_2_NH^-^) and the ligands were energy minimised (MMFF94x force field). All protein structures were obtained from the RCSB protein databank: hCA I (pdb: 3lxe, 1.90 Å), hCA II (pdb: 4e3d, 1.60 Å), hCA IX (pdb: 3iai; 2.20 Å) and hCA XII (pdb: 1jd0; 1.50 Å). The protein atoms and the active site zinc ions were retained and all other atoms were omitted. The remaining structure was protonated using the protonate 3 D functionality of MOE and subsequently, the obtained structure was energy-minimised (AMBER14:EHT)^32^. Finally, the obtained protein models were superposed on the hCA I structure using the backbone Cα-atoms and all Zn^2+^-ions, zinc-binding histidines and the overall backbone atoms superposed well (RMSD value: 1.281 Å). Docking calculations were performed using the FlexX docking tool (v2.3.2; BioSolveIT GmbH, St. Augustin, Germany) within MOE. The binding pocket was defined as all residues within 6.5 Å of the reference ligand acetazolamide. The sulphonamide tail of the ligands was forced to adopt a similar orientation and interactions to the Zn^2+^ ion as observed for acetazolamide using a pharmacophore model. All ligands were docked fifty times and the best scoring three poses were subjected to refinement calculations^33^. To this end, the ligand and binding pocket residues were energy minimised and rescored using GBVI/WSA force field^34^.

## 4. Carbonic anhydrase inhibition studies

All the tested compounds had no affinity for the common off-target hCA I isoform (*K*_i_ >100 µM) and some of them were more active against the tumour-related isoform hCA IX if compared with the parent drug 2-(benzylsulfinyl)benzoic acid (Table 1). The derivatives **1**–**3** bearing the ketone group and with benzyl moiety bound to the sulphur atom, exhibited affinity and selectivity against the tested isoforms depending on the sulphur atom oxidation state. Compound **1** exhibited better activity than the lead compound against hCA IX (*K*_i_ hCA IX = 1.1 µM) and was ineffective against the two off-targets (*K*_i_ hCA I/II >100 µM). The oxidation to sulfoxide (**2**) impaired the selectivity “restoring” the inhibitory activity owned by the lead compound for hCA II, although in the high micromolar range, while the affinity towards hCA IX (**2**, *K*_i_ hCA IX = 2.0 µM) was slightly inferior than **1**. The sulphone **3** displayed affinity exclusively for hCA IX, although weaker than **1** and **2** (**3**, *K*_i_ hCA IX = 16.4 µM).

**Table 1:** Inhibitory activity of derivatives **1**–**53** and the reference drug (acetazolamide, AAZ) against the four selected hCA isoforms by a stopped-flow CO_2_ hydrase assay^28^.

Compound	Structure	*K*_i_ (μM)^a^			
		hCA I	hСа II	hСа IX	hСа XII
**1**		>100	>100	1.1	nt
**2**		>100	63.2	2.0	nt
**3**		>100	>100	16.4	nt
**4**		>100	>100	21.8	nt
**(*R*)-5**		>100	0.21	1.4	nt
**(*S*)-5**		>100	0.093	1.2	nt
**6**		>100	>100	2.3	nt
**7**		>100	75.2	15.0	nt
**(*R*)-8**		>100	84.9	15.3	nt
**(*S*)-8**		>100	65.7	20.0	nt
**9**		>100	>100	11.4	nt
**10**		>100	45.7	1.0	nt
**(*R*)-11**		>100	>100	1.4	nt
**(*S*)-11**		>100	>100	18.1	nt
**12**		>100	40.1	21.1	nt
**13**		>100	>100	>100	nt
**14**		>100	>100	>100	nt
**15**		>100	>100	>100	nt
**16**		>10	8.22	0.046	2.66
**(*R*)-17**		>100	52.2	2.2	nt
**(*S*)-17**		>100	51.9	1.9	nt
**18**		>10	2.67	>10	0.066
**19**		>100	86.3	19.8	nt
**(*R*)-20**		>100	52.2	22.8	nt
**(*S*)-20**		>100	38.9	24.6	nt
**21**		>100	68.2	25.8	nt
**22**		>100	>100	>100	nt
**23**		>100	>100	>100	nt
**24**		>100	>100	6.5	>100
**25**		>100	>100	34.5	>100
**26**		>100	>100	14.0	>100
**27**		>100	>100	16.7	>100
**28**		>100	>100	40.9	>100
**29**		>100	>100	22.7	>100
**30**		>100	90.9	>100	>100
**31**		>100	>100	38.2	>100
**32**		>100	>100	2.3	>100
**33**		>100	>100	2.5	>100
**34**		>100	>100	36.4	>100
**35**		>100	>100	13.5	>100
**36**		>100	>100	2.7	>100
**37**		>100	>100	27.3	>100
**38**		>100	>100	8.7	>100
**39**		>100	>100	>100	>100
**40**		>100	>100	>100	>100
**41**		>100	>100	>100	>100
**42**		>100	>100	>100	>100
**43**		>100	>100	>100	>100
**44**		>100	>100	>100	>100
**45**		>100	>100	>100	>100
**46**		>100	>100	>100	>100
**47**		>100	>100	>100	>100
**48**		>100	>100	>100	>100
**49**		>100	>100	>100	>100
**50**		>100	>100	>100	>100
**51**		>100	>100	>100	>100
**52**		>100	>100	>100	>100
**53**		>100	>100	>100	>100
**AAZ**		0.25	0.012	0.25	0.006

a Mean from three different determinations (errors in the range of 5–10% of the reported values). nt: not tested.

The replacement of **1** methyl group with the hydroxyl one to give the **4**, the sulphide analogue of the lead compound, had detrimental effects reducing affinity for hCA IX (*K*_i_ hCA IX = 21.8 µM) but still conserving the selectivity (*K*_i_ hCAI/II >100 µM). The oxidation to sulfoxide ((*R*/*S*)**-5**) and sulphone (**6**) improved inhibitory activity in the low micromolar range against the tumour-related isoform. However, while **6** bearing the sulphonyl moiety was inactive towards the two off-target actings selectively against hCA IX (**6**, *K*_i_ hCA I/II > 100 µM; *K*_i_ hCA IX = 2.3 µM), the two sulphinyl enantiomers of **5** exhibited their preference for hCA II ((*R*)-**5**, *K*_i_ hCA II = 0.21 µM; (*S*)-**5**, *K*_i_ hCA II = 0.093 µM) rather than hCA IX ((*R*)-**5**, *K*_i_ hCA IX = 1.4 µM; (*S*)-**5**, *K*_i_ hCA IX = 1.9 µM). Comparing the inhibition data against hCA II of **5** enantiomers, it was quite clear the preference of the enzyme for (*S*)-**5**, which was the eutomer. Indeed, after incubation with the racemic mixture of **5**, only the adduct between hCA II and (*S*)-**5** was observed^20^. Compounds **7–9** were endowed with a methyl ester functional group, keeping constant the benzyl group bound to the sulphur atom (in the different oxidation states). These derivatives showed reduced inhibitory activity against hCA IX, regardless the sulphur oxidation number. Furthermore, compounds **7** and (*R*/*S*)-**8** were also weakly active against hCA II (65.7 < *K*_i_ hCA II (µM)<84.9). The unfavourable effect of the additional methyl group was mitigated by the insertion of a chloro atom at the *para-*position of the phenyl ring (**10**–**12**); this substitution counteracted the detrimental effects of the methyl group, probably enforcing other interactions inside the enzyme. Compound **10** was a good inhibitor of hCA IX exhibiting *K*_i_ against hCA IX of 1.0 µM, with a high micromolar residual activity against hCA II (*K*_i_ hCA IX = 45.7 µM). The oxidation to sulphinyl group ameliorated selectivity, because the two enantiomers (*R*/*S*)-**11** were ineffective against both of hCA I and II (*K*_i_ hCA I/II >100 µM). These two optical isomers showed different affinity towards hCA IX, with the (*R*)-enantiomer that inhibited this isoform better than the (*S*)-one ((*R*)-**11**, *K*_i_ hCA IX = 1.4 µM; (*S*)-**11**, *K*_i_ hCA IX = 18.1 µM). The further oxidation to sulphone (**12**) gave a detrimental effect, reducing both activity against hCA IX (*K*_i_=21.1 µM) and selectivity (*K*_i_ hCA II = 40.1 µM). The formal insertion of a carbonyl moiety between the methylene group and the phenyl ring in order to move the latter away from the sulphur atom, led to the **13**–**15** derivatives. The presence of this benzoyl moiety was not tolerated by this scaffold, giving compounds which did not exhibit effects towards all the tested isoforms (*K*_i_>100 µM).

The consequence of the amidic moiety insertion was also evaluated (**16–18**). Compound **16**, containing a non-oxidised sulphur atom, was the best inhibitor of the series against hCA IX displaying *K*_i_ of 0.046 µM. This result was slightly impaired by the oxidation to sulphinyl derivatives ((*R*)-**17**, *K*_i_ hCA IX = 2.2 µM; (*S*)-**17**, *K*_i_ hCA IX = 1.9 µM), while the corresponding sulphone (**18**) completely lost the affinity for hCA IX. All the amidic derivatives showed affinity for hCA II, albeit the sulphide (**16**) and sulphone (**18**) inhibited this isoform better than the sulphinyl enantiomers, which exhibited *K*_i_ values around 50 µM (**16**, *K*_i_ hCA II = 8.22 µM; **18**, *K*_i_ hCA II = 2.67 µM). The presence of a methyl group on the amidic moiety was detrimental for the inhibitory activity towards hCA IX. Indeed, the derivatives **19**–**21**, endowed with monomethyl amide, were less effective against the cancer-related isoform (19.8 < *K*_i_ hCA IX (µM) <25.8) than the analogues containing primary amide. Furthermore, the activity against hCA II was also lowered in the high micromolar range (36.9 < *K*_i_ hCA II (µM)<86.3). This behaviour was very similar to that observed for molecules containing a methyl ester moiety, which were less effective compared with their carboxylic acid analogues.

The effect and the importance of the phenyl moiety were also challenged by substituting it with linear alkyl chains of increasing length (**22–38**) and testing the influence against the two off-target hCA I and II, and the two tumour-related isoforms hCA IX and XII. The first attempts were done with the two compounds **22** and **23**. These derivatives, bearing methyl ester with the sulfoxide group linked to butyl and pentyl chain respectively, were inactive against all the tested isoforms, underlying the detrimental effects of this structural combination. However, better results were obtained when the amide moiety was placed instead of methyl ester one (**24–38**). The derivatives **24–38** displayed their effect exclusively against hCA IX, without any inhibitory activity against hCA I, II and XII. Only for derivative **30**, bearing propyl chain bound to the sulphur atom, we observed activity against hCA II, but with a very low affinity (*K*_i_ hCA II = 90.9 µM). Compound **32**, the sulphone analogue of sulphide **30**, was the best inhibitor against hCA IX among the compounds substituted with alkyl chains, inhibiting the cancer-related isoform with a *K*_i_ value of 2.3 µM. Other good results were found with **33** and **36**, containing a sulphur atom and bearing butyl or pentyl chain, respectively (**33**, *K*_i_ hCA IX = 2.5 µM; **36**, *K*_i_ hCA IX = 2.7 µM). Except for derivatives with propyl chain (**30–32**), the compounds containing the non-oxidised sulphur atom showed better inhibitory activity than the related sulfoxide and sulphone analogues (Table 1). In order to evaluate the role and the importance of sulphur atom in this scaffold, we performed its bioisosteric replacement with methylene moiety and oxygen or nitrogen atoms (**39–53**), keeping constant the functional groups constituting the scaffold seen above (e.g. carboxylic acid, amide, etc.). Some of the obtained compounds showed structures completely comparable to the lead compound (see for example **39, 41**). As one can see from Table 1, all the attempts done changing the sulphur atom were ineffective, giving compounds unable to inhibit all the tested isoforms. Furthermore, also the influence of the tested functional groups like the acidic or amidic one, were useless, enforcing and underling the importance that sulphur atom has for the activity of this scaffold. In light of the above, some considerations can be done. Among the functional groups tested (carboxylic acid, methyl ester, etc.) the best hCA IX inhibitor was provided with amidic moiety, although ketone and carboxylic acid derivatives also showed good activity. The insertion of a methyl group on the carboxyl acid or the amide impaired activity. This could be due to the altered H-bond donator/acceptor profile, or most likely to the increased steric hindrance. From all the data evaluated, it was quite clear that the oxidation of sulphur atom did not influence the activity or selectivity in a specific manner. Furthermore, in most of the efforts done, the sulphide derivative was the best inhibitor of hCA IX among the sulphide/sulfoxide/sulphone analogues endowed with the same substituent. The only exception was for carboxylic acid derivatives, whose better activity was exhibited by the sulphinyl ones.

The chiral resolution of the single sulfoxide enantiomers did not give a specific trend, with few enantiomers exhibiting better activity compared with the related optical isomer. The benzyl moiety was essential for the inhibition and the presence of halogen on the ring improved activity when compared with the unsubstituted analogues. The replacement of phenyl ring with linear alkyl chains impaired the activity, while raising the selectivity. The benzoyl moiety was not tolerated by this scaffold, affording compounds ineffective against all the tested isoforms. These outcomes could be related to the loss of hydrophobic interactions due to the phenyl ring shift, clashes with amino acids owing to the ring new position, or both of them. Nevertheless, the reasons of these data might be other. In fact, the inserted “spacer” (the carbonyl group), has an own steric hindrance along with specific bond angles and a stable dipole; all these features could affect negatively the interaction with the isoform IX. Finally, the substitution of the sulphur atom with oxygen, nitrogen one, or methylene underlined the importance of the sulphur atom for the activity of these compounds, because all the obtained derivatives were inactive against all the tested isoforms. Furthermore, some selected compounds were also tested against hCA XII to preliminary provide information on this target. All the derivatives, except compounds **16** and **18**, were inactive. Sulphone **18** was a potent and selective hCA XII inhibitor with *K*_i_ 0.066 µM, whereas sulphur-based **16** was a medium-potency inhibitor.

## 5. Molecular modelling studies

### 5.1. Docking studies into the active site of hCA IX

**Figure 2.**
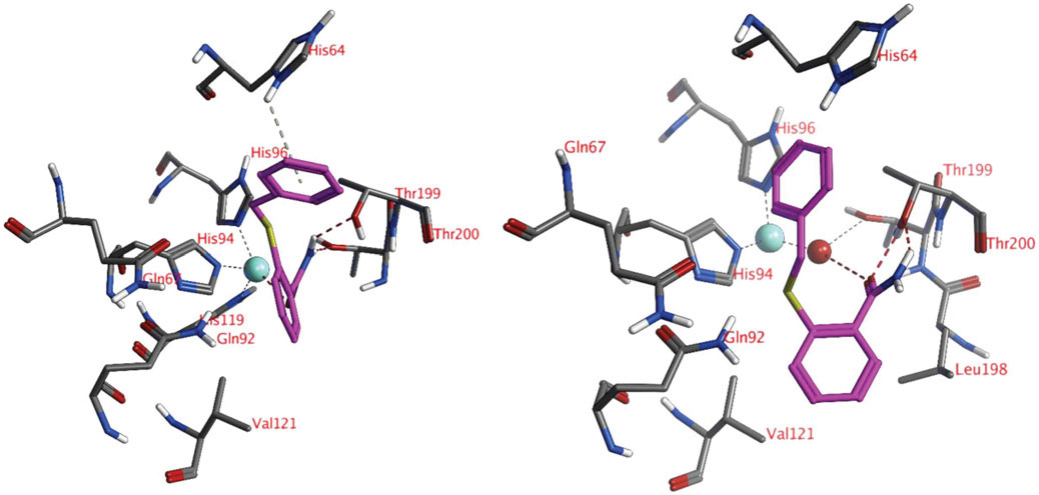
The docked poses of compound **16** (purple) in the active site of hCA IX forming an interaction with either the active site Zn^2+^-ion (panel **A**) or the zinc-bound water molecule (panel **B**). Zn^2+^ is indicated with a turquoise sphere, the water molecule is indicated with a red sphere, hydrogen bonds and interactions to the Zn^2+^-ion are indicated in red dashed lines and arene-H interactions are indicated in yellow dashed lines.

The lowest *K*_I_ value for hCA IX was measured for compound **16** (*K*_i_ = 46 nM), which is lower compared to the reference compound acetazolamide (*K*_i_ = 250 nM). Two different binding interactions with the hCA IX active site were suggested. In the first docked pose, the ligand directly formed an interaction with the Zn^2+^-ion via its carbonyl group (Figure 2(A)). The amine group of the ligand formed hydrogen bonds with the side chain hydroxyl groups of Thr199 and Thr200. One of the ligand’s phenyl group interacted with the side chain of His64 (arene-H interaction). Changing the amino group or the phenyl group or replacing the S atom by SO or SO_2_ groups made it more difficult for the ligands to adopt this pose. Several compounds (including compounds **1**, **5**, **10**, **11**, **24** and **32**) can also adopt poses in which a direct interaction with the Zn^2+^ is possible. In the second docked pose, the ligand carbonyl group formed a hydrogen bond to the zinc-bound water molecule (Figure 2(B)). In addition, the carbonyl and amine group of the ligand formed hydrogen bonds with the side chain hydroxyl group of Thr200. Hydrophobic interactions were formed with the side chains of His94 and Val121. The replacement of the amino group with a hydroxyl group or the replacement of the S atom by SO or SO_2_ groups may be tolerated according to this pose. The later may form hydrogen bonds with the side chain of Gln92. Furthermore, many compounds can adopt docked poses in which an interaction with the water molecule is possible.

### 5.2. Docking studies into the active site of hCA XII

**Figure 3.**
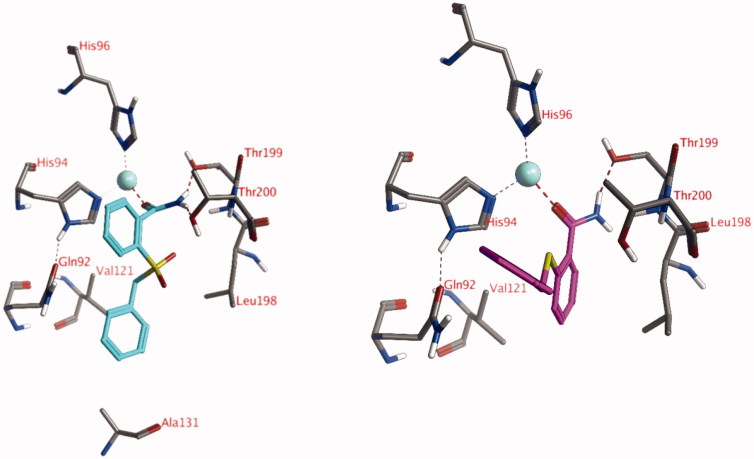
The docked poses of compound **18** (turquoise, left) and compound **16** (purple, right) in the active site of hCA XII forming interactions with the active site Zn^2+^-ion. Zn^2+^ is indicated with a turquoise sphere, hydrogen bonds and interactions to the Zn^2+^-ion are indicated in red dashed lines.

The lowest *K*_i_ value for hCA XII was measured for compound **18** (*K*_i_ = 66 nM) and the second-lowest *K*_i_ value was measured for compound **16** (*K*_i_ = 2.66 µM). Two docked poses have been obtained for these compounds: both formed an interaction with the Zn^2+^-ion via the ligand carbonyl groups (Figure 3). The amino group of compound **18** formed hydrogen bonds with the side chains of Thr199 and Thr200, while the amino group of compound **16** only established a hydrogen bond to the side chain of Thr199. Both ligands formed hydrophobic interactions with His94. In addition, compound **18** established a hydrophobic interaction with the side chain of Val121. No poses have been obtained for these compounds with an interaction with the zinc-bound water molecule.

### 5.3. Docking studies into the active site of hCA I and hCA II

Although the actives sites of the investigated hCA isozymes are very similar to each other, important differences still exist that most likely influence the binding interactions of the ligands. In hCA I, His200 is present instead of Thr200 and therefore this hydrogen bonding opportunity (see Figures 2 and 3) was no longer present and the ligands cannot approach the Zn^2+^-ion to form direct interactions or interactions with a zinc-bound water molecule. This may be responsible for the low activity of these compounds against hCA I. Similarly, a relatively large Phe131 is present in hCA II instead of Ala131 in hCA XII. As such, the docked pose as observed of compound **18** in the active site of hCA XII (Figure 3(A)) would not be possible due to a clash with Phe131.

## 6. Conclusion

In conclusion, we have explored the chemical space of the main functional groups of the 2(benzylsulfinyl)benzoic acid, an innovative and atypical hCA inhibitor. We have designed, synthesised, and tested 53 derivatives against the most important hCA isoforms establishing robust SARs within this scaffold. Most of them were selective hCA IX inhibitors in the low micromolar/high nanomolar range. The stereochemistry of the sulfoxide group had no impact on the biological activity, whereas the isosteric replacement of the sulphur atom with oxygen, nitrogen or methylene led to a total loss of activity. Molecular modelling studies demonstrated different binding poses of the inhibitors within the active site, differently from that observed for the parent compound.
